# Does ALK-rearrangement predict favorable response to the therapy of bevacizumab plus pemetrexed in advanced non-small-cell lung cancer? Case report and literature review

**DOI:** 10.1186/s40169-017-0178-x

**Published:** 2018-01-09

**Authors:** Zhichao Liu, Youting Bao, Butuo Li, Xindong Sun, Linlin Wang

**Affiliations:** 1 School of Medicine and Life Sciences, University of Jinan-Shandong Academy of Medical Sciences, Jinan, 250200 China; 2 Department of Radiation Oncology, Shandong Cancer Hospital Affiliated to Shandong University, Shandong Academy of Medical Science, No. 440, Ji Yan Road, Jinan, 250017 Shandong China; 3 Clinical College, Weifang Medical University, Weifang, 261053 China

**Keywords:** ALK, ALK inhibitor resistance, Non-small cell lung cancer, Pemetrexed, Bevacizumab

## Abstract

**Background:**

Advanced ALK-rearranged non-small cell lung cancer (NSCLC) patients will develop acquired resistance after anaplastic lymphoma kinase (ALK) inhibitors therapies. Vascular endothelial growth factor-A (VEGF-A) production and tumor vessel formation were found to be more significantly enriched in ALK-rearrangement NSCLC than that in epidermal growth factor receptor or Kirsten rat sarcoma viral oncogene mutated NSCLC. However, the correlation between ALK rearrangement and the efficacy of bevacizumab (a recombinant humanized IgG1 monoclonal antibody targeting VEGF-A) was still elusive.

**Case presentation:**

We report a case with metastatic NSCLC harboring ALK-rearrangement who was initially resistant to two courses of ALK-Tyrosine Kinase Inhibitor (TKI) therapy, but got a clinical benefit of 7 months of progression free survival after the combined treatment of bevacizumab plus pemetrexed. And the patient tolerated well.

**Conclusions:**

It suggested that bevacizumab combined with pemetrexed might be a preferred option for ALK rearrangement patient who had failed no less than two courses of ALK-TKIs.

## Background

Patients diagnosed as anaplastic lymphoma kinase (ALK) rearrangement comprise 3–7% of non-small cell lung cancer (NSCLC) cases [1]. Rearrangements of the ALK gene lead to an oncogene addicted state due to the aberrant ALK activation. Three generations of ALK target inhibitors, including crizotinib, ceritinib, alectinib and lorlatinib, have been developed and used in targeted therapy; and ALK positive patients could get longer progression free survival (PFS) and better objective response rate (ORR) of 53–65% compared to the cellular toxic chemotherapy [2–6]. However, almost all patients will develop acquired resistance inevitably. Thus, the option of next therapeutic strategies is problematic in particular with regard to its insensitivity to various standard chemotherapies.

Bevacizumab targeting tumor angiogenesis showed encouraging efficacy as the first-line therapy for patients with advanced non-squamous NSCLC [7]. Epidermal Growth Factor Receptor (EGFR) mutation-positive patients had a significantly longer PFS on bevacizumab compared with wild type EGFR patients in NSCLC [8]. However, the correlation between ALK rearrangement and the efficacy of bevacizumab was still elusive. Besides, it has been showed that patients with ALK-positive tumor status seemed to have a better prognosis when treated with pemetrexed [9]. And adding pemetrexed to bevacizumab was associated with a significant PFS benefit compared with bevacizumab alone in patients with non-squamous NSCLC. However, few studies have focused on the combination of bevacizumab with pemetrexed in ALK-positive patients.

Here, we presented a case with ALK positive lung adenocarcinoma getting significant clinical benefit from bevacizumab to pemetrexed combination therapy who had failed two courses of ALK-inhibitor therapy.

## Case presentation

**Fig. 1:**
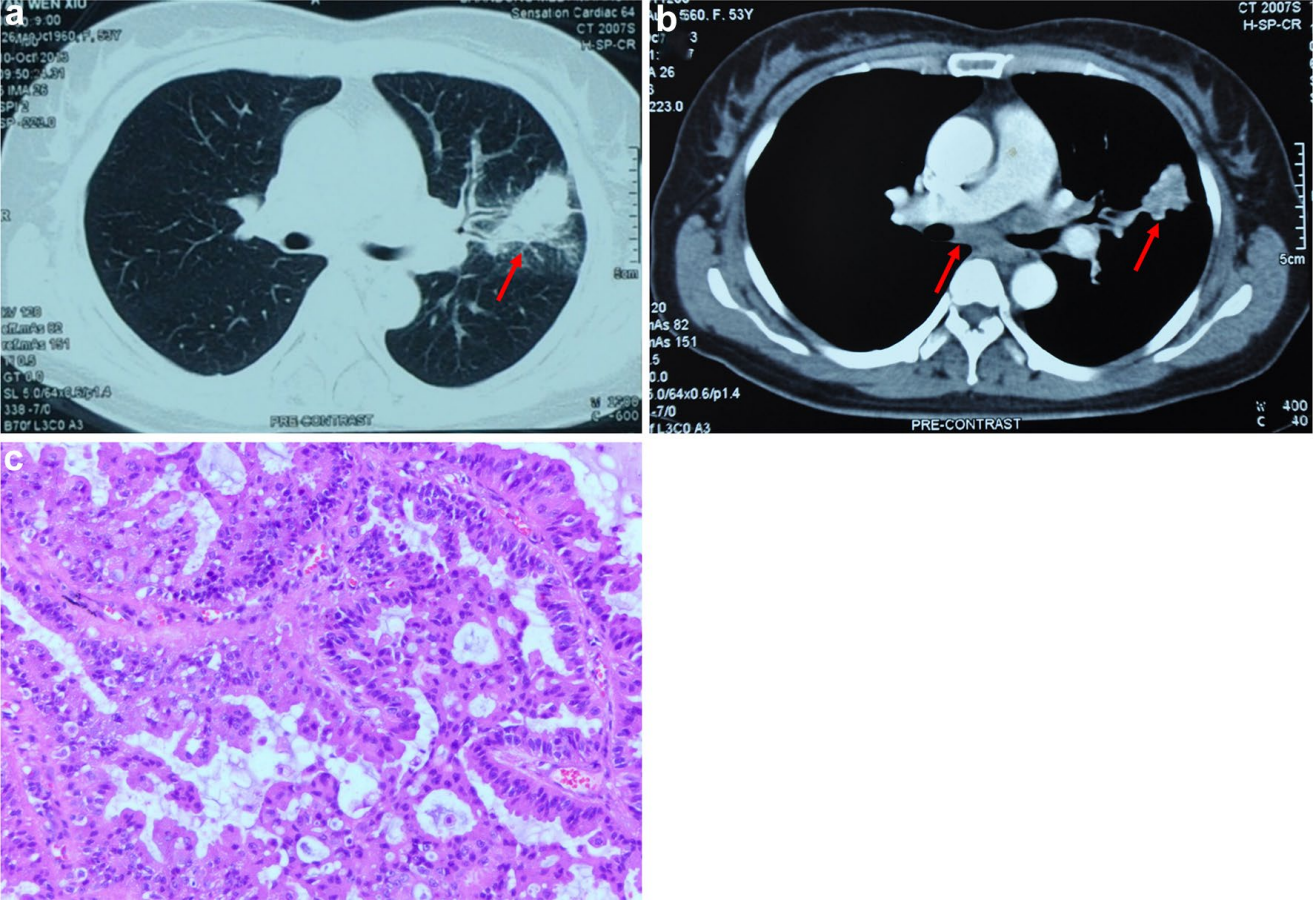
The Chest CT images and histology of the left lung neoplasm. **a** Pulmonary window revealed a single pulmonary nodule on the lower left lobe. **b** Mediastinal window revealed the single pulmonary nodule on the lower left and enlarged subcarinal lymph nodes. **c** Hematoxylin and eosin staining demonstrated adenocarcinoma (magnification×200)

A 55-year-old Asian never-smoker female presenting with an irritable dry cough for a month was examined in a community hospital in October 2013. Computed tomography (CT) scan of chest revealed a single pulmonary nodule (approximately 2.5 × 5 cm) on the lower left lobe and enlarged subcarinal lymph nodes (Fig. 1a, b). No metastases in brain, liver, bone and so on were found. After biopsy of the left lung lesion, she was diagnosed as lung moderately differentiated adenocarcinoma (Fig. 1c) and the stage was IIIA (cT2N2M0).

**Fig. 2:**
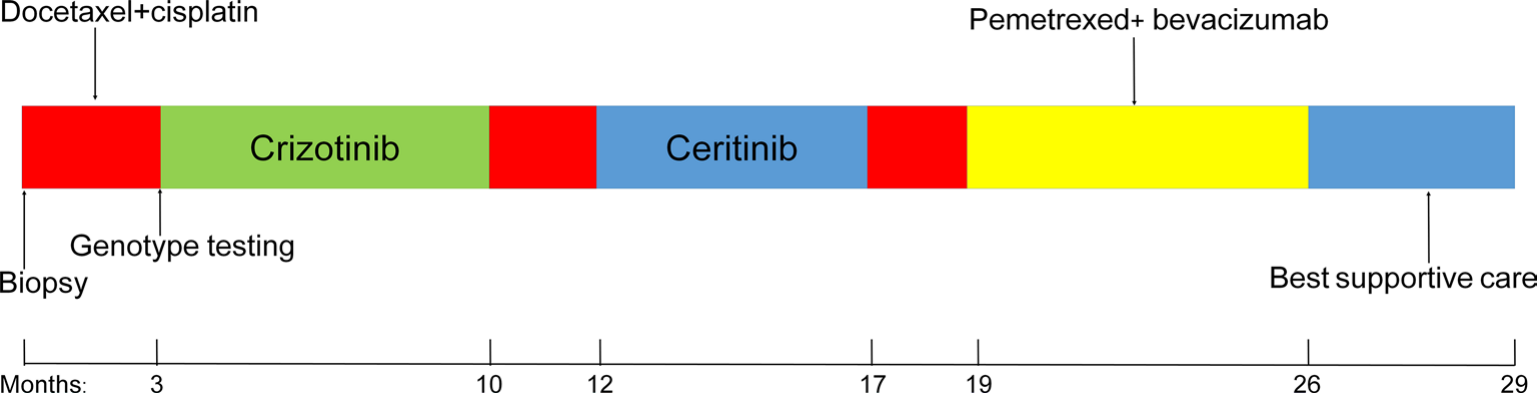
Timeline of treatment shows the administration of multiple therapeutic approaches

**Fig. 3:**
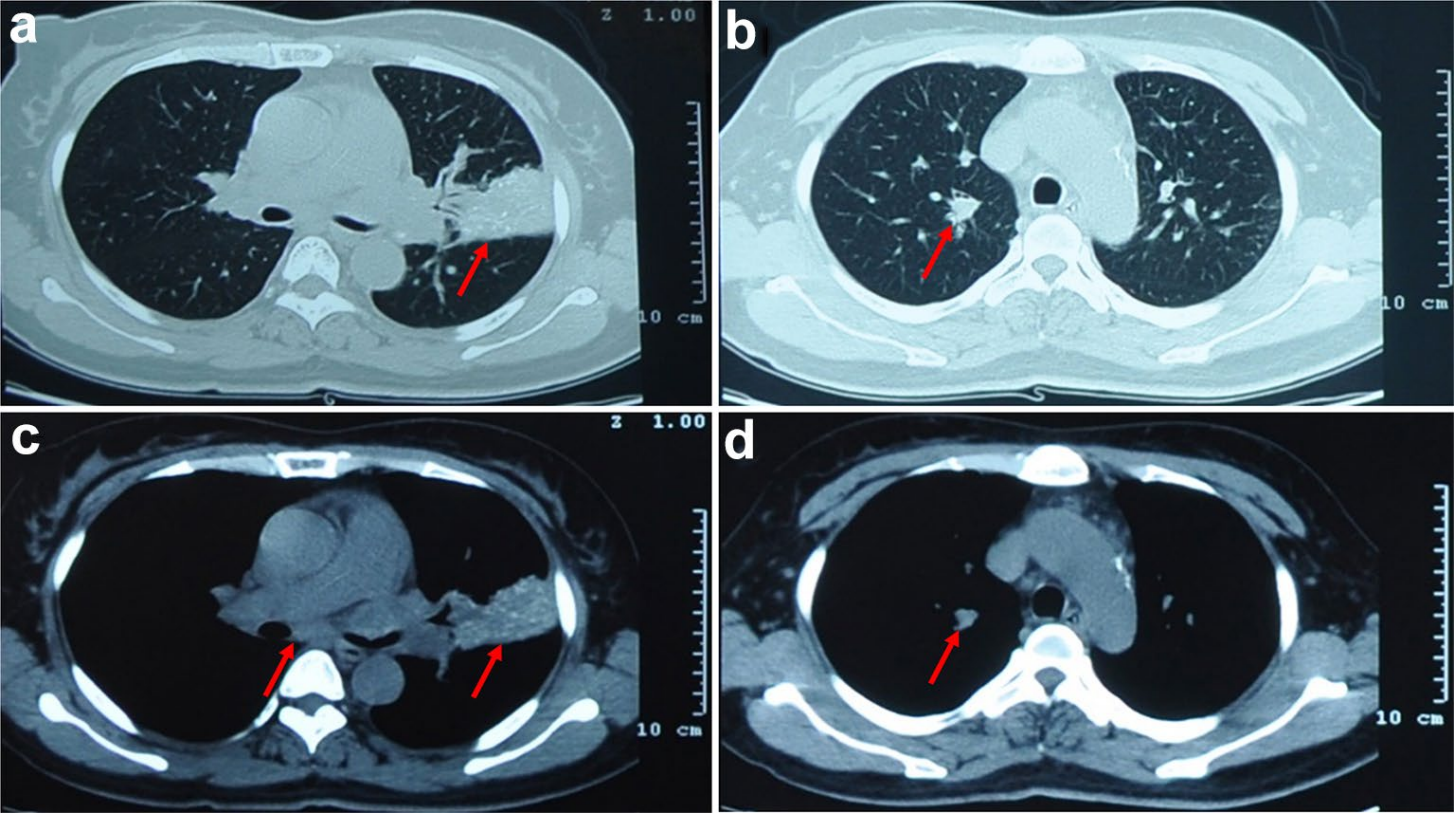
Imaging examination confirmed the disease progression after two cycle inducing chemotherapy. **a**, **c** The primary lesion on the lower left lobe was larger than before. **b**, **d** New metastasis in the right upper lobe was found

**Fig. 4:**
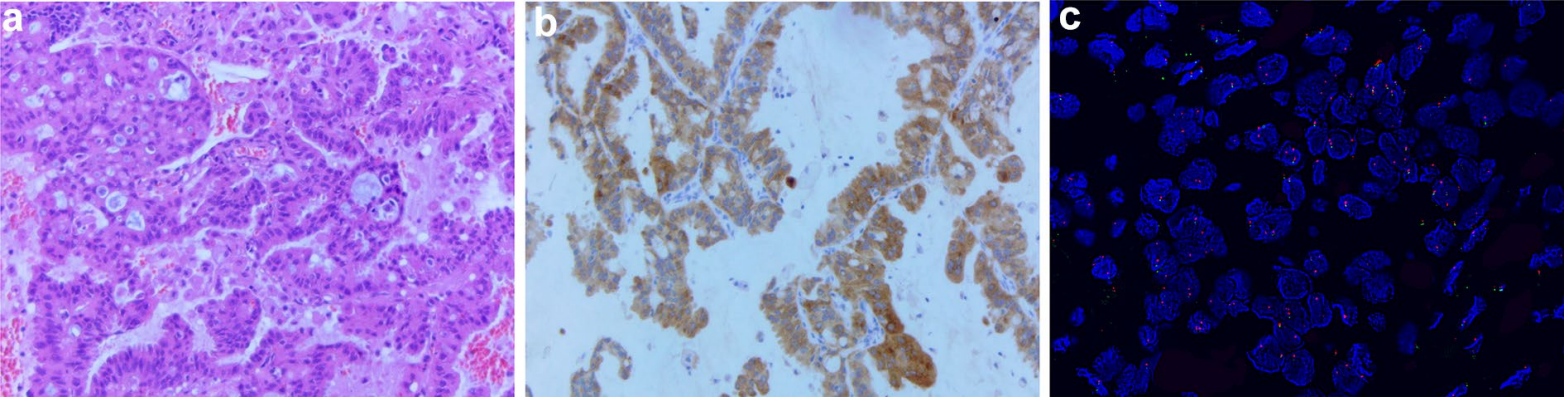
Re-evaluation and genotype testing of the left lung neoplasm. **a** Hematoxylin and eosin staining demonstrating adenocarcinoma (magnification×200). **b** Immunohistochemical staining for the ALK protein revealing strong granular cytoplasmic expression in the lung adenocarcinoma specimen (magnification×200). **c** EML4-ALK rearrangement positive confirmed by fluorescence in situ hybridization (magnification×1000)

She was recommended for two cycles of inducing chemotherapy with docetaxel (75 mg/m^2^ D1 − D1 = D21) and cisplatin (75 mg/m^2^ D1 − D1 = D21) and then the concurrent radiotherapy and chemotherapy (Fig. 2). However, the primary lesion on the lower left lobe was larger and new metastasis in the right upper lobe was found after these two cycle chemotherapy (Fig. 3). Re-evaluation and genotype testing of the left lung lesion showed no EGFR mutation, but luckily, the strong expression of ALK (ventana); and EML4-ALK gene fusion was positive by fluorescence in situ hybridization (Fig. 4).

**Fig. 5:**
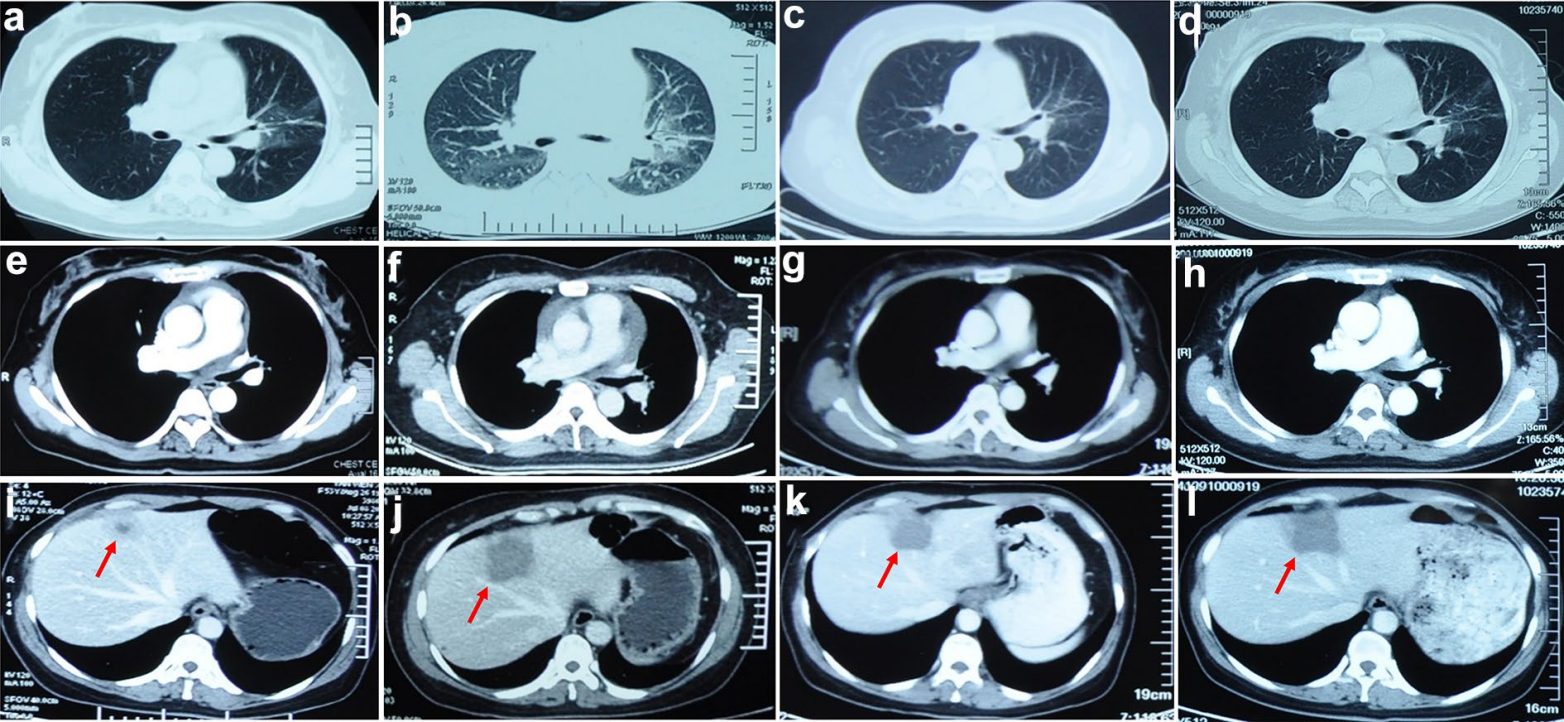
Disease evaluation during the treatment of crizotinib and ceritinib. **a**–**h** The CT scan revealed complete remission of lung lesions. **i** A single liver nodule was found after the 6 months of starting crizotinib. **j** The enlarged liver lesions was revealed on 20 Aug. 2014. **k** The liver metastasis shrinked after the treatment of ceritinib. **l** The liver lesions enlarged again after the 5 months of starting ceritinib

The patient received crizotinib treatment (250 mg, bid, orally) starting from 7 Jan. 2014 (Fig. 2). The primary lesion on the lower left lobe and metastasis in the right upper lobe was disappeared 1 month later. Stable condition maintained until 30 Jun. 2014 when metastatic nodules were found on liver (Fig. 5a, e, i). Microwave ablation was used to treat with these metastatic nodules. And crizotinib was continued until 20 Aug. 2014, when liver lesions enlarged again (Fig. 5b, f, j).

Then the patient began to receive ceritinib (750 mg, qd, orally) from 8 Oct. 2014 (Fig. 2); 1 month later, the liver metastasis shrinked apparently (partial response, PR) (Fig. 5c, g, k). However, adverse events including acute liver function lesion (CTCAE) (grade 2/CTCAE ver. 4.0) and severe diarrhea occurred. The ceritinib dose was then decreased from 750 to 450 mg. Although there were no other metastases, the treatment was discontinued because of liver metastasis progression in March 2015 (Fig. 5d, h, l).

**Fig. 6:**
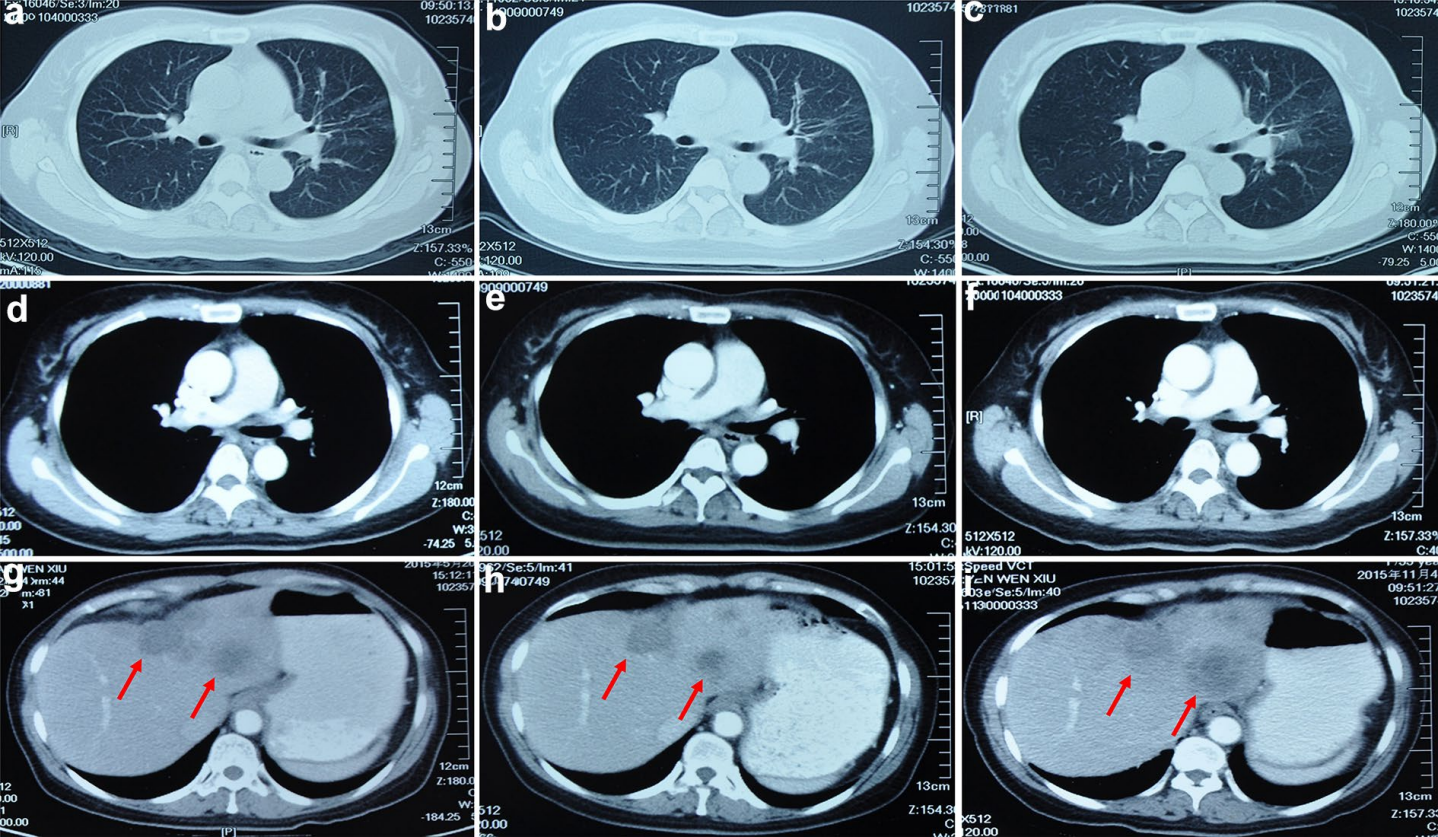
Disease evaluation during the treatment of pemetrexed with bevacizumab. Before treatment of pemetrexed with bevacizumab, liver metastases were assessed using CT scan and no lung lesions were found (**a**, **d**, and **g**). After two cycle therapy of pemetrexed with bevacizumab, metastatic nodule size in liver decreased (partial response) (**b**, **e** and **h**). The CT scan showed the disease progression in liver metastases after eight cycle administration of pemetrexed plus bevacizumab (**c**, **f** and **i**). During the entire course, there were still no metastases in bilateral lungs, mediastinum, brain and so on

The patient came to our hospital on 4 May 2015 (Fig. 6a, d, g). After the re-biopsy of liver metastasis, three EML4–ALK resistance mutations (C1156Y, D1203N and L1198F) was found. The patient began to receive treatment of pemetrexed (500 mg/m^2^ D1 − D1 = D21) with bevacizumab (5 mg/m^2^ D1 − D1 = D21) from 11 May 2015 (Fig. 2). After two cycle treatment, metastatic nodule size in liver decreased, and there were no new metastases in bilateral lungs, brain and so on, which demonstrated the disease of PR (Fig. 6b, e, h). After four and six cycle treatment, the CT scan both revealed stable disease. The patient tolerated well and the performance status is 1. Following additional two cycle of pemetrexed with bevacizumab, the liver metastatic tumor showed radiographic progression by the CT scan on 4 Dec. 2015 (Fig. 6c, f, i). The best supportive care was administrated, and the patient ultimately died of liver failure in March 2016 (Fig. 2).

## Discussion

ALK rearrangement, a distinctive subset of NSCLC, is associated with several distinctive clinical and pathologic features including younger in age, never/light smoking with adenocarcinoma, men in gender, more likely to have abundant signet ring cells and absence of EGFR and Kirsten rat sarcoma viral oncogene (KRAS) mutations [10]. Crizotinib has been acknowledged as standard first line option for ALK-rearranged NSCLC, demonstrating a response rate of 60–74% and a median PFS of 7–11 months based on the “PROFILE” clinical trial program (Table 1) [5, 6, 11–18]. Besides, second generation ALK-inhibitors, ceritinib or alectinib, also have shown a significantly superior median PFS versus second line traditional chemotherapy (pemetrexed or docetaxel) when the patients have the progressed disease (Table 1) [5, 6, 11–18]. However, patients will almost inevitably relapse and the therapeutic options are thus more limited after the treatment of first and second generation ALK-inhibitors.

**Table 1:** Trials about the efficacy of first- and second-generation ALK inhibitors

Study	Phase of study	Treatment	Number of patients	ORR (%)	PFS (months)	Patient population	References
PROFILE 1001	I	Crizotinib	149	60.8	10	125 of 149 ALK rearranged patients had disease progression on ≥ 1 chemotherapy	[5]
PROFILE 1005	II	Crizotinib	261	60	8.1	ALK rearranged patients had disease progression on ≥ 1 chemotherapy	[6]
PROFILE 1007	III	Crizotinib vs docetaxel or pemetrexed	173 174	65 20	7.7 3.0	ALK rearranged patients previously treated with chemotherapy (platinum doublet)	[11]
PROFILE 1014	III	Crizotinib vs pemetrexed +cispaltin/carboplatin	172 171	74 45	10.9 7.0	Treatment-naive ALK rearranged patients	[12]
ASCEND3	II	Ceritinib	124	63.7	11.1	ALK rearranged patients ALK inhibitor-naive but had disease progression on chemotherapy.	[13]
ASCEND4	III	Ceritinib vs pemetrexed +cispaltin/carboplatin	189 187	72.5 26.7	16.6 8.1	Treatment-naive ALK rearranged patients	[14]
ASCEND5	III	Ceritinib vs docetaxel or pemetrexed	231 116	39.1 6.9	5.4 1.6	ALK rearranged patients previously treated With chemotherapy (platinum doublet) and crizotinib	[15]
J-ALEX	III	Crizotinib vs alectinib	104 103	70.2 85.4	10.2 20.3—not reached	Treatment-naive ALK rearranged patients	[16]
NP28763	II	Alectinib	138	50	8.9	ALK rearranged patients previously treated with chemotherapy and crizotinib	[17]
NP28761	II	Alectinib	87	52.2	8.1	ALK rearranged patients previously treated with chemotherapy and crizotinib	[18]

There are multiple mechanisms of resistance to the second generation ALK-inhibitors [19]. Mutations in the ALK tyrosine kinase domain are the main reason for the resistance, which have been identified in approximately one-third of the re-biopsied tumors of these resistant patients [20]. Among these mutations, G1202R is the most common mutation type found in patients progressing on the second-generation ALK-tyrosine kinase inhibitor (TKI) [19]. Lorlatinib, an extremely selective ALK-TKI with activity also targeting ROS1 kinase, has been shown to overcome resistance mediated by the ALK G1202R secondary mutation in preclinical study. More importantly, in a phase I/II study, lorlatinib has demonstrated ORR of 46% and PFS of 9.2 months in patients pretreated with more than two courses of ALK-TKIs [2, 21]. Besides, ALK inhibitor therapy also could be tailored dependent on the variable types of mutation (Table 2) [13, 21–35]. However, treatment with lorlatinib seems very expensive in developing countries and selection of ALK-TKI based on patients’ mutation type still lacks strong clinical evidence.

**Table 2:** The resistant mutations and sensitive mutations of ALK inhibitors

ALK-TKI	Sensitive mutations	Insensitive mutations	References
Cizotinib	L1198F	1151Tins, L1152P, L1152R, C1156Y, I1171T, F1174C, F1174L, F1174V, L1196M, G1202R, S1206, G1269, G1269S, R1275Q	[21–24]
Ceritinib	I1171T, L1196M, S1206Y, G1269A, F1174L, V1180L	C1156Y, 1151Tins, L1152R, F1174C, G1202R, G1123S	[25–28]
Alectinib	1151Tins, L1152R, C1156Y, F1174L, F1174V, L1196M, S1206Y, G1269A, R1275Q	I1171T, I1171S, V1180L, G1202R	[28–32]
Lorlatinib	F1147L, 1151Tins, L1152R, C1156Y, L1192R, L1196M, G1202R, S12026Y, G1269A	L1198F	[22, 33–35]

Pemetrexed-based chemotherapy may be another option for patients progressing on the second generation ALK-TKI. The PFS with pemetrexed based therapy for ALK-rearranged NSCLC patients is significantly longer than in patients without ALK rearrangements or with either EGFR or KRAS mutant [36–38]. Besides, pemetrexed was shown to be superior to docetaxel in both ORR (29% vs. 7%, respectively) and PFS (4.2 months vs. 2.6 months, respectively) for ALK-rearrangement NSCLC patients progressing on platinum-based chemotherapy [5]. All of these implied that pemetrexed should be preferentially considered for the treatment of ALK-rearrangement lung adenocarcinoma. However, the overall prognosis of patients with ALK-rearrangement NSCLC was still not encouraged.

Bevacizumab shows encouraging efficacy as the first or second-line therapy for patients with non-squamous NSCLC in some studies [39]. The phase III BEYOND trial compared the efficacy of carboplatin/paclitaxel plus placebo and carboplatin/paclitaxel plus bevacizumab in a Chinese patient population, and showed that the median OS was extended by 6.6 months and the median PFS was extended by 2.7 months, respectively. In particular, the median PFS was 12.4 months in EGFR mutation positive tumors and 8.3 months in wild-type tumors after the carboplatin/paclitaxel plus bevacizumab treatment [8]. However, the correlation between ALK rearrangements and the efficacy of bevacizumab remain unanswered. Besides, angiogenesis was also found to play an important role in the biology of ALK rearranged NSCLC. It has been shown that ALK-positive patients have higher levels of vascular endothelial growth factor-A (VEGF-A) and tumor vessel formations compared to EGFR and KRAS mutated NSCLC [40]. Moreover, treatment with the anti-VEGF-A antibody bevacizumab strongly impaired Anaplastic Large Cell Lymphoma (containing EML4-ALK rearrangement) growth in mouse xenografts [41].

In addition, several previous clinical reports have also indicated the potential benefits of bevacizumab on advanced ALK-rearrangement NSCLC. For example, adding bevacizumab to chemotherapy was found to effectively control radioresistant brain metastases in an ALK-rearrangement lung adenocarcinoma patient [42]. Treated with bevacizumab combined chemotherapy, lung nodule of ALK-rearrangement NSCLC showed significant shrinkage after two cycles of therapy, and the PFS or OS has not been reached after 8 cycles of treatment [43]. In another retrospective study, prolonged responses (18 months) were observed after treatment of weekly paclitaxel and bevacizumab in the ALK-rearrangement NSCLC patient [44]. Finally, long-term disease control was also observed after treatment with pemetrexed and bevacizumab in NSCLC patients with ALK or ROS1 gene rearrangements compared with general nonsquamous NSCLC population [45].

Here, we presented a case with ALK positive lung adenocarcinoma getting clinical benefit of 7 months of PFS from bevacizumab to pemetrexed treatment after two failed courses of ALK-inhibitor therapy; and the toxicity was well tolerated. It seems that addition of bevacizumab to pemetrexed treatment tend to be a favorable option for the resistant ALK-rearrangement NSCLC.

However, there are still some questions to be addressed in the near future. First, at present, the patients in the “PROFILE” and “ASCEND” trials were administrated standard chemotherapy or ALK-TKI as the first or second line therapies. However, none study is being planned in order to compare bevacizumab combined with chemotherapy versus ALK-TKI in the treatment-naïve or previously treated ALK-rearrangement NSCLC patients. Second, given the effectiveness of bevacizumab and ALK-TKI in the ALK-rearrangement NSCLC, trials of NCT02521051 and NCT02946359 combing these two kinds of drugs are being undertaken [46, 47]. Third, although the patient in our report got 7 months of PFS in the fourth line treatment, the efficacy of bevacizumab plus pemetrexed for ALK-rearrangement NSCLC is still needed to be studied in future prospective trials. ALK-rearrangement may predict favorable response to the therapy of bevacizumab plus pemetrexed in advanced non-small-cell lung cancer.

## Conclusion

We present this case with ALK-rearrangement metastatic NSCLC who was initially resistant to two courses of ALK-TKI therapy, but got a clinical benefit from the combined treatment of bevacizumab plus pemetrexed and we conduct a review of the related literature. It demonstrates that ALK-rearrangement may predict a favorable response to the therapy of bevacizumab combined with pemetrexed in advanced non-small cell lung cancer and this combination may be a reasonable choice for advanced ALK-rearrangement NSCLC patient when ALK-TKIs treatment failed. Further studies are still needed to confirm the efficacy of bevacizumab plus pemetrexed for ALK-rearrangement NSCLC.

## Abbreviations

ALK: anaplastic lymphoma kinase
NSCLC: non-small cell lung cancer
VEGF-A: vascular endothelial growth factor-A
PFS: progression free survival
TKI: Tyrosine Kinase Inhibitor
ORR: objective response rate
EGFR: Epidermal Growth Factor Receptor
CT: computed tomography
CEA: carcinoembryonic antigen
PR: partial response
KRAS: Kirsten rat sarcoma viral oncogene
